# Trends in diabetes incidence in Austria 2013–2017

**DOI:** 10.1038/s41598-023-35806-0

**Published:** 2023-05-29

**Authors:** Michaela Kaleta, Michael Leutner, Stefan Thurner, Gottfried Endel, Noemi Kiss, Martin Robausch, Peter Klimek, Alexandra Kautzky-Willer

**Affiliations:** 1 Section for Science of Complex Systems, CeMSIIS, Medical University of Vienna, Spitalgasse 23, 1090 Vienna, Austria; 2 Complexity Science Hub Vienna, Josefstädter Straße 39, 1080 Vienna, Austria; 3 Unit of Gender Medicine, Clinical Division of Endocrinology and Metabolism, Department of Internal Medicine III, Medical University of Vienna, Währinger Guertel 18–20, 1090 Vienna, Austria; 4 Santa Fe Institute, 1399 Hyde Park Road, Santa Fe, NM 85701 USA; 5 Austrian Social Insurance (Dachverband der Sozialversicherungen), Kundmanngasse 21, 1030 Vienna, Austria; 6 Austrian Health Insurance Fund (Österreichische Gesundheitskasse), Kremser Landstraße 3, 3100 St. Pölten, Austria; 7 Gender Institute, Julius Kiennast-Strasse 79, 3571 Gars am Kamp, Austria

**Keywords:** Diabetes, Diabetes, Epidemiology

## Abstract

This study aims to quantify whether age and sex groups in Austrian regions are equally affected by the rise of type 2 diabetes. Population-wide medical claims data was obtained for citizens in Austria aged above 50 year, who received antihyperglycemic treatments or underwent HbA1c monitoring between 2012 and 2017. Diabetes incidence was measured using an epidemiological diabetes progression model accounting for patients who discontinued antihyperglycemic therapy; the erratic group. Out of 746,184 patients, 268,680 (140,960 females) discontinued their treatment and/or monitoring for at least one year. Without adjusting for such erratic patients, incidence rates increase from 2013 to 2017 (females: from 0·5% to 1·1%, males: 0·5% to 1·2%), whereas they decrease in all groups after adjustments (females: − 0·3% to − 0·5%, males: − 0·4% to − 0·5%). Higher mortality was observed in the erratic group compared to patients on continued antihyperglycemic therapy (mean difference 12% and 14% for females and males, respectively). In summary, incidence strongly depends on age, sex and place of residency. One out of three patients with diabetes in Austria discontinued antihyperglycemic treatment or glycemic monitoring for at least one year. This newly identified subgroup raises concern regarding adherence and continuous monitoring of diabetes care and demands further evaluation.

## Introduction

Many European countries are facing a growing prevalence of type 2 diabetes. An estimated 7–11% of the Austrian population suffer from diabetes, an estimate increasing with time^1^. Next to reduced quality of life, diabetes often causes severe complications such as cardiovascular disease, renal failure, blindness, neuropathies or amputation. Prevalence of diabetes and its complications is expected to increase even further due to the ageing population, resulting in a severe and growing burden for both individual patients and health care systems.

Recent research found regional differences in diabetes prevalence within and across several countries^2,3^. For instance, for Germany regional heterogeneity is captured within the Diabetes Atlas^4^ or within the DIAB-CORE study^5^ on a national level.

Until recently, exact data on diabetes prevalence were missing in Austria which still lacks a national registry for adults with diabetes. Current information is derived from local registries and fragmented datasets, health surveys are based on data from drug prescription or from preventive medical check-ups^1^. The most recent report of the International Diabetes Federation estimated, based on in-country data sources for adults aged 20–79 years with diabetes, a number of 447.1 thousand and an age-adjusted comparative prevalence of 4.6%^6^ which is lower than that shown in the last report, partly due to different data sources and methodology. An estimation of 5.9% diagnosed and 3.6% undiagnosed cases was reported in 2015^1^; however these estimations were derived from a health survey in 2007. A more recent evaluation of screening tests in Austria revealed a decreasing trend in newly diagnosed diabetes cases to 1.8% in 2016^1^. Data on incidence is generally scarce. Reliable data on incidence are only available for patients up to 14 years, almost all type 1 diabetes, in Austria, based on a national registry for children.

Here we use a nation-wide claims dataset collected by the umbrella organization of social security institutions in Austria, which covers 99.9% of all residents in Austria. As part of its performance monitoring system (“LEICON”), the insurances maintain a database combining inpatient and outpatient claims data (drug dispensals, hospitalizations, physician visits, etc.) of all insured patients receiving antihyperglycemic medications or related treatments as identified from a prescribed algorithm (see Methods), covering the years 2012–2017. This dataset opens up the possibility to measure levels and trends in diabetes prevalence and incidence in specific patient subcohorts with unprecedented precision. Therefore the aim was to identify increases and decreases in diabetes prevalence, incidence and treatment in Austria based on epidemiological modeling, taking age and sex differences into account.

## Research design and methods

### Data description

We used a pseudonymised research dataset provided by the LEICON group within the Austrian Health Insurance Fund (Österreichische Gesundheitskasse)^1,7,8^. This dataset was constructed from population-wide inpatient and outpatient administrative claims data which includes all patients aged 50 years and older meeting any of the following inclusion criteria (as defined by the LEICON group), namely any antihyperglycemic drug prescriptions (using Anatomical Therapeutic Chemical (ATC) codes) or at least two claims for blood glucose and HbA1c measurements in the Austrian insured population between 2012 and 2017.

**Figure 1:**
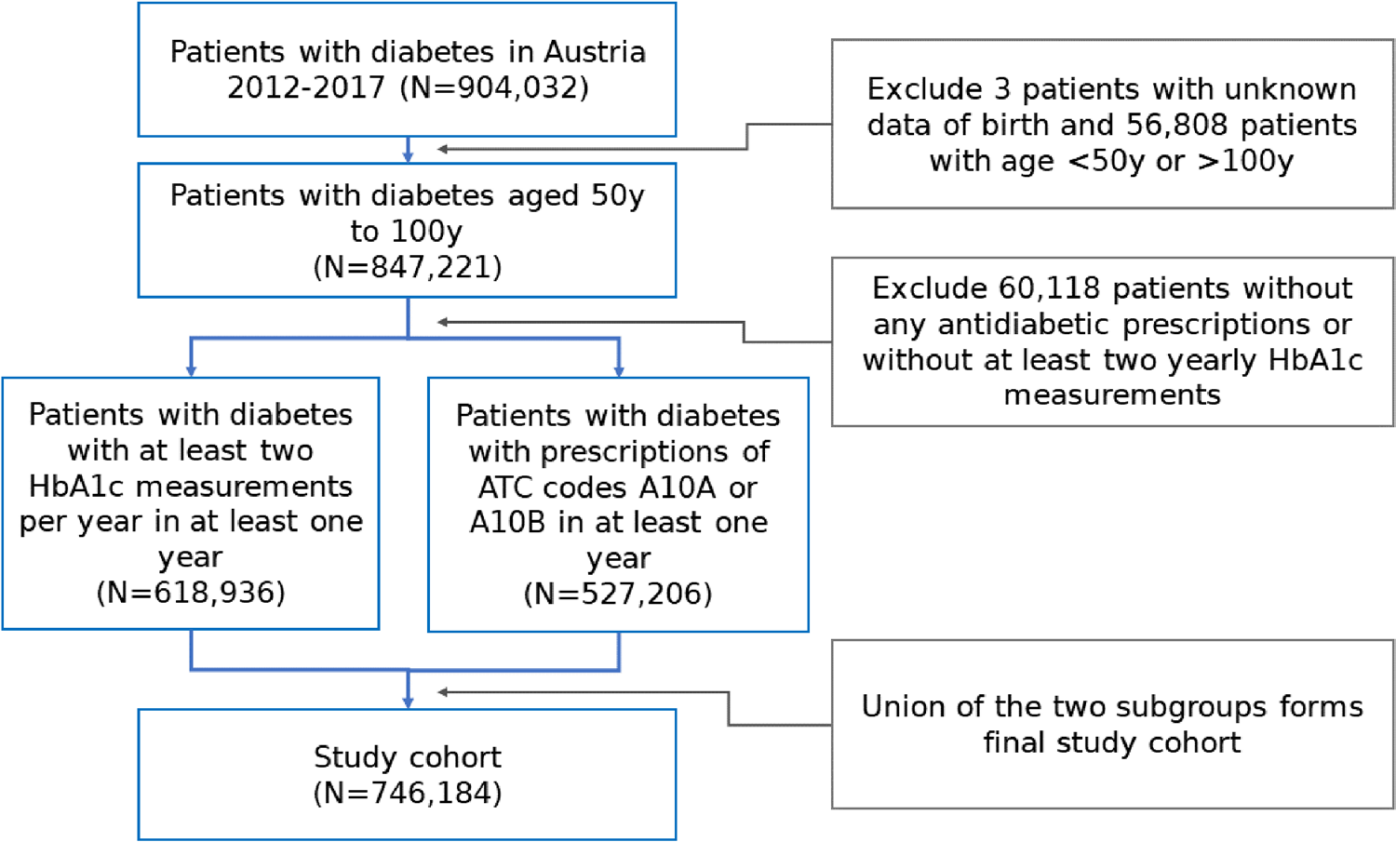
Cohort description. Patient selection and exclusion criteria for final study cohort based on patients’ prescribed medication and HbA1c measurements.

Information on date of birth and death, sex and district of residence as well as date and dose of all antihyperglycemic medications (ATC codes starting A10) and HbA1c measurements were obtained. Furthermore, general population information required for model calibration was taken from the Austrian statistical office (Statistik Austria). In each dataset, age was measured at the beginning of each calendar year. Information on comorbidities was derived from main and side diagnoses (10th revision of the International Statistical Classification of Diseases and Related Health Problems (ICD-10)) associated with hospital stays during the entire observation period. Figure 1 shows inclusion and exclusion criteria starting from the original research dataset (N = 904,032) until final study cohort (N = 746,184).

### Epidemiological model

We use a compartmental modelling approach (“NIDEX model”) on the level of political districts (administrative regions with a mean population of 77,000) to measure age- and sex-specific diabetes prevalence and incidence rates. Each year each patient can be in one of the following compartments or states: non-diabetes (N), incident (I), diabetes (D), erratic (E), or deceased (X); see Supplemental Figure S1. Patients that are not captured in the LEICON dataset are classified as N(on-diabetes). In the first year, in which a relevant claim is recorded for a patient, they are moved from the N compartment to the I(ncident) compartment. From then onward, they are also member of the D(iabetes) compartment and stay there as long as they fulfill at least one inclusion criterion each year. If, however, a patient had one calendar year without fulfilling any of the inclusion criteria, they are classified as E(rratic). Finally, there is a state “deceased” that patients enter when they die. Prevalence can be measured over time via the stocks (number of patients in a given state) whereas incidences and mortality rates via the flows between different stocks. There are four different flows that may affect the stock of patients with diabetes, (i) new or incident diabetes patients (patient flow $${D}^{In}$$), (ii) patients who discontinued their treatment ($${E}^{D}$$), (iii) patients with diabetes that continue their treatment after suspending it for more than one year ($${D}^{E})$$ and (iv) death ($${X}^{D}$$). All patient states and the transitions of patients between them can be observed in the data without the need for additional assumptions. Incidence rates for males and females were measured as a function of age within this model; see Supplemental Material for detailed information. An alternative model that does not contain erratic patients, the NIDX model, is also described there. Incidence and prevalence rates for age groups were obtained as harmonic means over the respective rates in each age group. This analysis was considered a relevant sensitivity analysis because higher incidence rates may ensue in the NIDX model if erratic patients are counted as being incident multiple times, compared to the NIDEX model, where they are only counted once.

Ethics approval and consent to participate was not applicable as this is a modelling study based on the re-use of an anonymized research dataset. All features that would allow the identification of individuals have been sufficiently aggregated to make such an identification only possible with disproportionate and unrealistic efforts. The Federal Law on Documentation in the Health Care System in Austria provides the legal basis for this study: It allows the documentation of health-related data in the intra- and extramural outpatient and inpatient care sectors, as well as for the processing of patients' and service providers' data in pseudonymized form for certain purposes including (long-term) monitoring of epidemiological developments relevant to health policy as well as the implementation and further development of integrated health structure planning and health services research.

## Results

Table 1 and Supplemental Table S1 show characteristics of the two cohorts of patients with diabetes and erratic patients. 187,142 females (4.3% of entire female population, mean age 65y, SD 12y) and 186,541 males (4.5% of entire male population, mean age 62 y, SD 11 y) were without diabetes in 2012 but incident in any following year. In the following, the dynamics of these cohorts in the observation period and their age-, sex-, birth-year-dependent as well as regional differences in incidence are reported.

**Table 1:** Descriptive cohort table.

	Patients with diabetes		Erratic patients	
	Females	Males	Females	Males
N	230,654	246,850	140,960	127,720
Age 2012 (mean ± SD)	69 ± 12	65 ± 11	68 ± 12	65 ± 11
Insulin therapy (ATC A10A)	57,534 (25%)	63,583 (26%)	6,281 (5%)	6,311 (5%)
Oral antihyperglycemic therapy (ATC A10B)	180,901 (78%)	200,626 (81%)	47,704 (34%)	48,876 (38%)
≥ 2 yearly HbA1c measurements	189,951 (82%)	200,376 (81%)	122,478 (87%)	110,669 (87%)

Sex-specific information on patients with diabetes and erratic patient cohorts aggregated over all years. Patients may appear in one or more therapy groups (based on prescribed ATCs A10A, A10B and number of HbA1c measurements).

### Dynamics of cohorts

**Figure 2:**
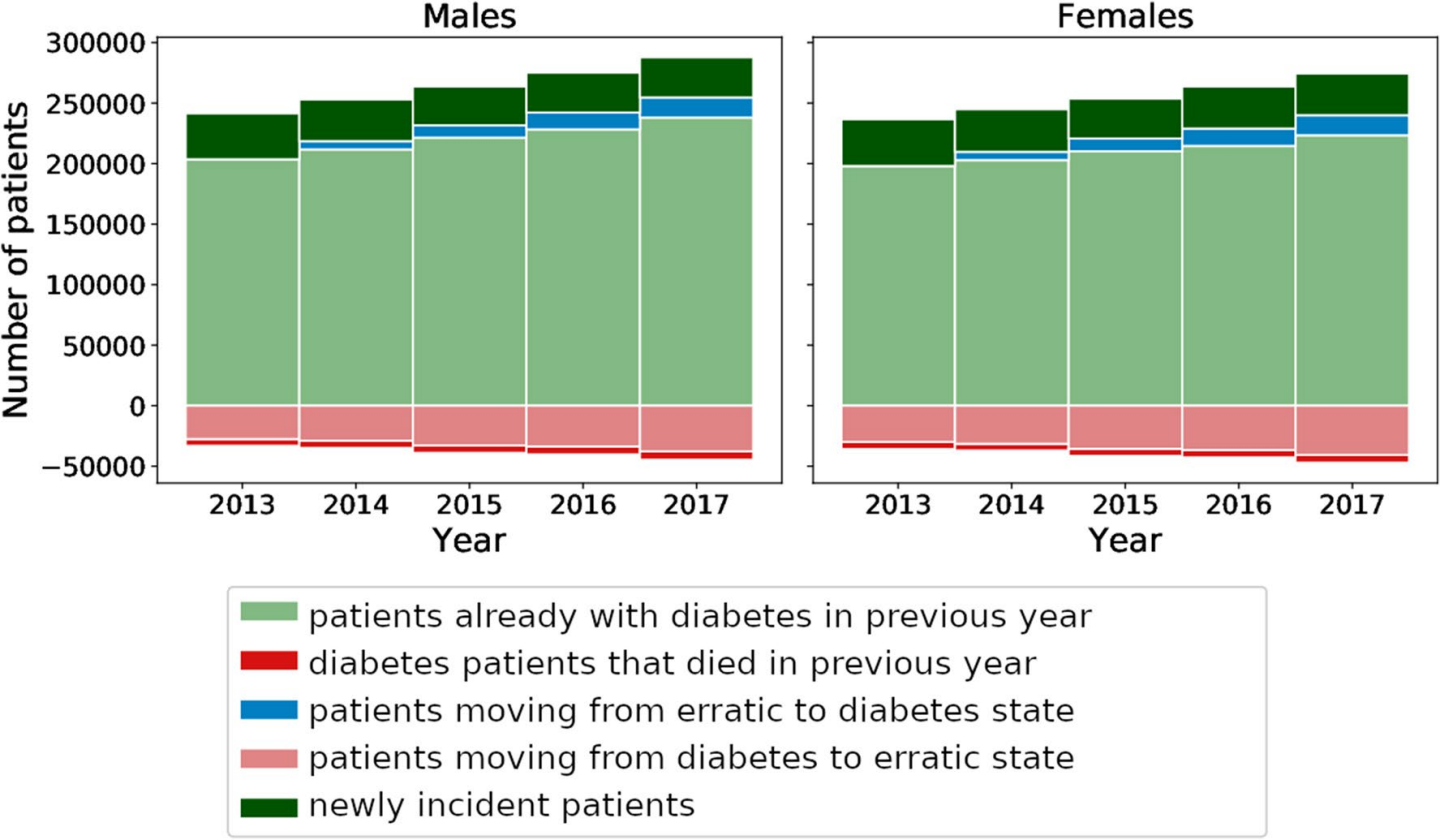
Composition of patients with diabetes over time. By summing ages 50 to 100 we show the stock of patients with diabetes and its in- and out-flows between 2013 and 2017 for males (left) and females (right). Inflows to the patient stock with diabetes are counted as positive while outflows are shown as negative. By construction, the first erratic patients enter the diabetes state in 2014 and their numbers grow until 2017.

We introduced two different types of cohorts with diabetes, patients who received continuous antihyperglycemic treatments (patients with diabetes) and those who discontinued their treatment for at least one year (erratic patients). Figure 2 shows the stock of patients with diabetes and its out- and inflows from other patient states for all females and males over the years 2013 to 2017. The prevalence ${D}$ increases from 198,119 in 2012 (11.7% of the total female population aged >  = 50 y) to 223,502 in 2016 (12.3%) for females and from 203,685 in 2012 (14.3%) to 238,222 in 2016 (15.1%) for males. Around 32,000 male and 35,000 female patients discontinue antihyperglycemic treatment and thereby switch to the erratic state each year.

**Figure 3:**
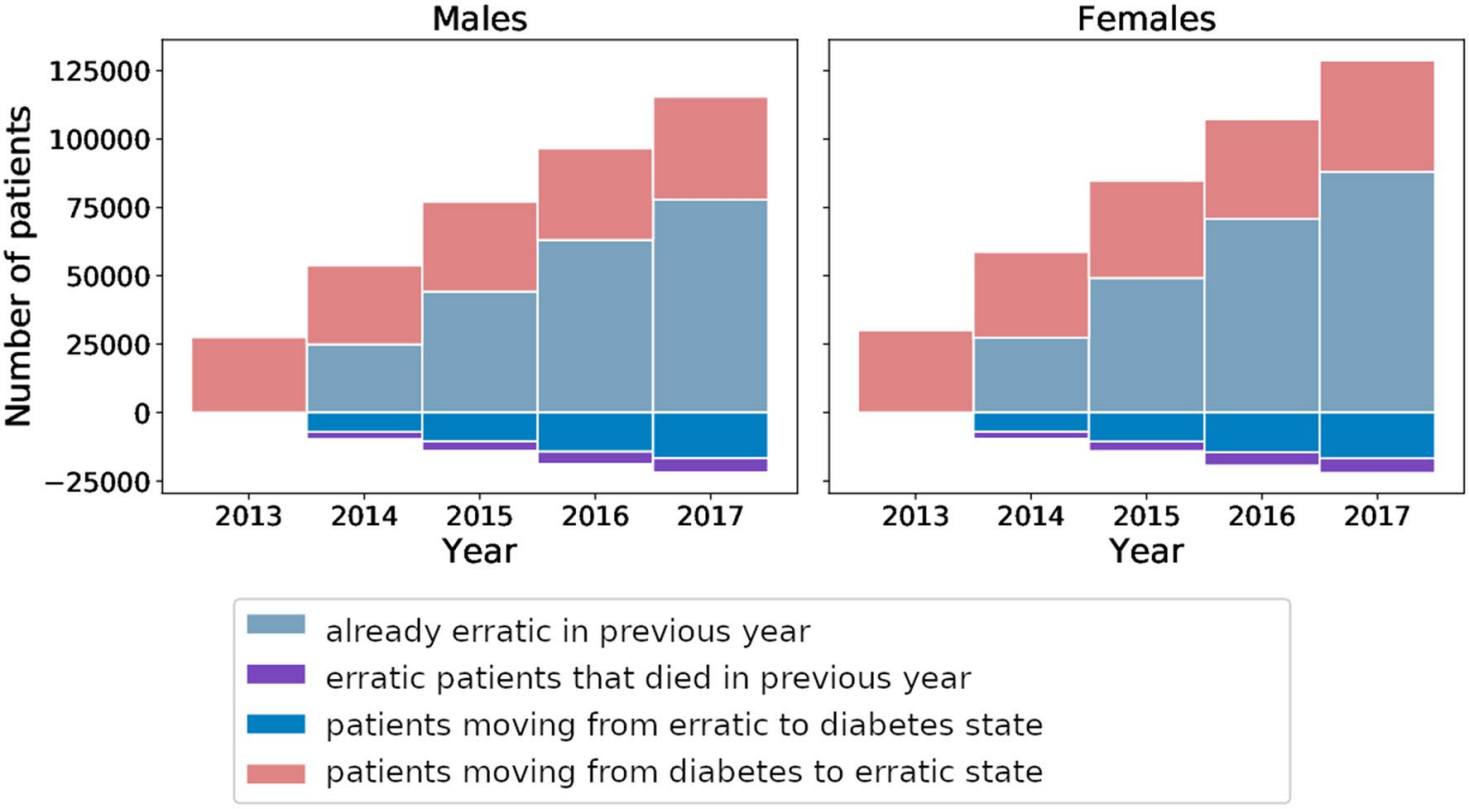
Composition of erratic patients. The stocks of erratic patients are summed over ages 50 to 100 for males (left) and females (right). Inflows to the erratic patients are shown as positive increments, outflows as negative ones. The first erratic patients who left the diabetes state appear in 2013. From 2014 onwards the first patients might resume their antihyperglycemic treatments, consistent with Fig. 2.

The dynamics of erratic patients are shown in Fig. 3. With each additional year, there is an increased chance that patients resume their antihyperglycemic treatments, with almost 17,000 such male or female patients in 2017, respectively. Nevertheless, the stock of erratic patients grows each year, reaching about 200,000 (6% of the total population aged >  = 50 y) erratic patients in 2017.

When comparing the mortality rate of patients with diabetes and erratic patients over all years and ages using the mean difference (MD), higher mortality rates both for erratic males (MD = 14%, *p*-value < 0.01) and erratic females (MD = 12%, *p*-value < 0.01) are observed.

### Changes in incidence over time

**Figure 4:**
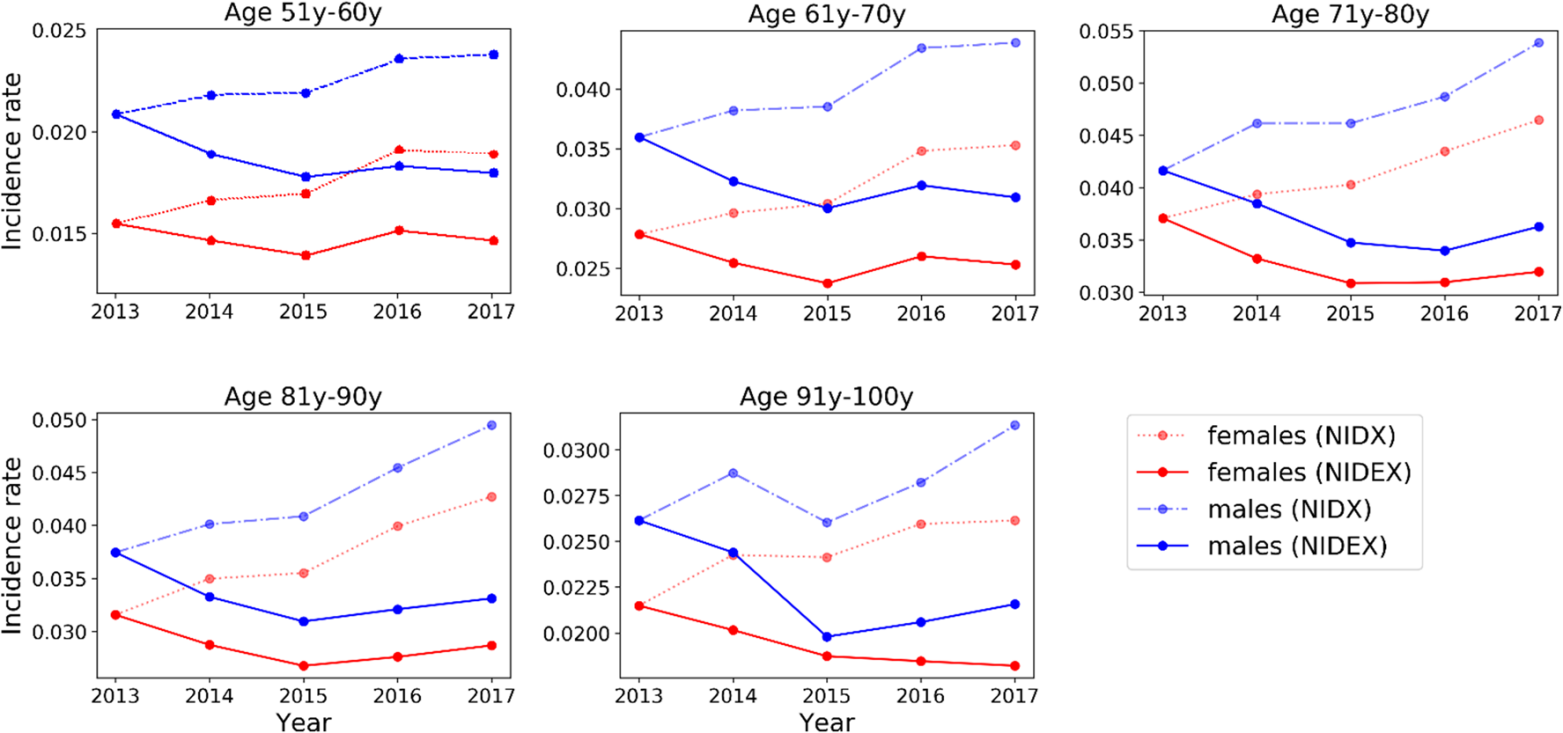
Temporal changes in incidence rates. Results are grouped into age groups of 10 years for female and male population and for analysis adjusting for patients discontinued treatments (NIDEX) and without such adjustments (NIDX), respectively. Overall there is a trend of decreased incidence rates between 2013 and 2017 in all age and sex groups. If erratic patients are not adjusted for, increasing incidence in all age and sex groups would be observed.

To measure changes of the incidence rate, age $a$ was categorized into age groups of 10-year intervals for both sex, see Fig. 4. In the NIDEX framework adjusting for patients with discontinued treatments, we see trends of decreasing incidence rates in all sex and age groups between 2013 and 2015, followed by inconsistent trends between 2015 and 2017. However, the overall trend for the incidence rate $\alpha$ between 2013 and 2017 is always decreasing (max. -0.5% females, -0.5% males). This is in contrast to measurements that do not adjust for erratic patients in the NIDX model. Without adjustments, incidences would be increasing over time (max. 1.1% females and 1.2% males) in all age and sex groups.

### Regional variation of type 2 diabetes

**Figure 5:**
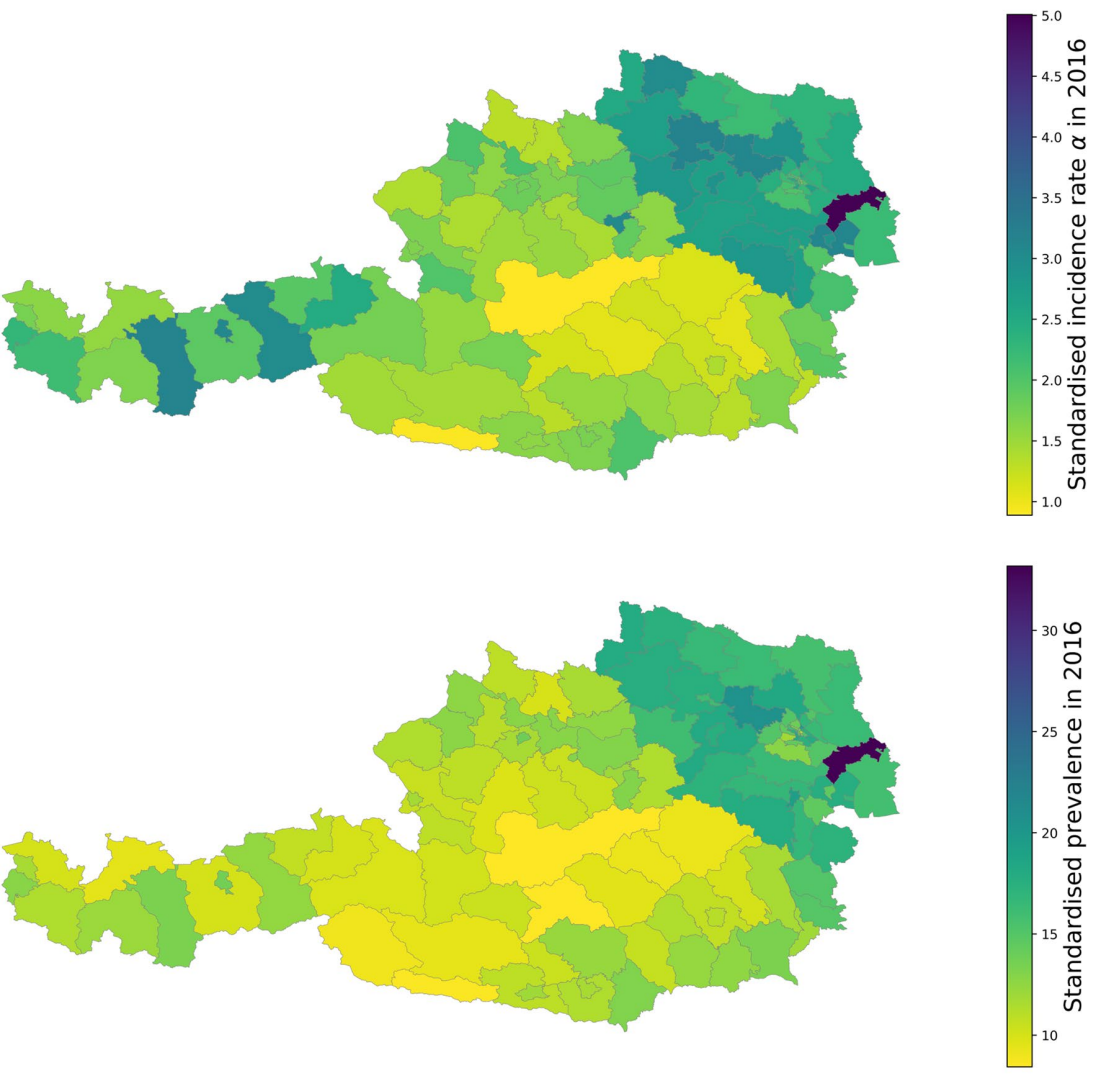
Regional variation of diabetes incidence (top) and prevalence (bottom) in Austria in 2016. Each district is colored depending on its age and sex standardised incidence (prevalence) rate. Light colors in central parts of Austria show districts with low incidence rates while darker colors mostly in the north east districts indicate higher incidence rates. The East–West gradient for the incidence is not as pronounced as for the prevalence, suggesting that some Austrian regions are “catching up” in terms of prevalence. The maps were created in Python v3.9 using the matplotlib v3.0.3 (https://matplotlib.org/) library.

The geographic distributions of incidence and prevalence rates in the Austrian population from 2016, standardized to age and sex, are shown in Fig. 5. There are substantial regional variations in diabetes incidence, varying between 1% (central parts of Austria) to more than 5% (Eastern-most regions). Vienna, the only Austrian city with a population exceeding 1 million inhabitants, shows incidence and prevalence that is not substantially different compared to the surrounding, more rural areas. A number of Western regions with low prevalence show higher incidence than most Southern or some Eastern regions, suggesting a catch-up growth in prevalence. Overall, diabetes prevalence and incidence rate $\alpha$ show a high correlation (Pearson’s correlation 0.830, see Supplemental Table S2).

## Discussion

There is a substantial number of patients in Austria who discontinue their antihyperglycemic treatment for at least a year. It can be assumed that the group without drugs but regular monitoring corresponds to patients being on lifestyle therapy only. Even if we expect these subjects to adhere to a healthy lifestyle, diet and exercise to manage their glycemia, the fact that glycemic monitoring, including regular HbA1c measurements, was discontinued, is concerning. The standards of diabetes care recommend HbA1c testing at least two times a year in patients who are meeting treatment goals, with stable glycemic control^9^. Patients who do not meet their glycemic goals or with therapy modifications should have quarterly tests. This fact is even more alarming as these patients with discontinued monitoring and/or drug therapy (erratic patients) also showed a significantly higher mortality than patients with regular or continuous care. Although neither the causes of mortality in our patients are known nor causality between discontinued management and mortality is evidenced, we cannot exclude some kind of association from a clinical point of view.

These erratic patients complicate the measurement of diabetes incidence and prevalence rates. Classifying them as incident leads to an increase in incidence in all age and sex groups from 2013 to 2017, whereas properly accounting for these patients results in a decrease of incidence over the same time. In this work we therefore present a model-based approach which ensures that these erratic patients with discontinued treatments are not erroneously counted as incident more than once.

Standardized incidence rates vary by a factor of up to 5 across individual districts. Regional differences in diabetes prevalence were previously described with higher prevalence in the East of the country, as well as differences based on sex, education and occupation^1,10^. The current analysis confirms regional differences in prevalence rates across Austria and reports incidence broken down in districts in Austria for the first time. The incidence map reveals clusters of high incidence in the west of the country in the last years. Higher rates are usually seen in urban areas with the highest rates in the metropolis Vienna and the surrounding areas. This East–West gradient may be due to a less healthy lifestyle, lower socioeconomic situation and higher migration background of the population in the East^11^. Diabetes was independently predicted by geographic location, psycho-social stress, lack of physical activity and age in both sexes in Austria in these previous analysis. However, trends may be changing as it appears in the current analysis that there are new clusters of high incidence in the West that deserve careful surveillance.

The Austrian health survey 2014 (Statistik Austria) reported a somewhat lower diabetes prevalence (4.7% vs. 5.5%) and incidence than that of 2007. Incidence rate was derived from medical preventive check-ups and showed a slight decrease from 2.3% to 1.8% of newly diagnosed diabetes cases. Incidence estimates were calculated from new prescriptions of antihyperglycemic drugs in comparison to one year before that date. Therefore, all these previous estimations relied on partial information and could not give a comprehensive picture of diabetes prevalence and incidence in Austria.

In the current study, a consistent increase in diabetes prevalence rates was found, which could be ascribed to better diagnosis and longer life expectancy of patients with diabetes in Austria in the last decades. In other countries including the US, prevalence rates remained at high levels in the last years while incidence rates even showed a trend toward decrease, but displaying great variability^2,3^. Interestingly, in Germany, based on analysis of national insurance data, a decrease in incidence rates was recently estimated for men and women by 2.4 and 1.7% per year between 2014 and 2019 with some regional variations^12^. However the authors observed an increase of the incidence rate in the population between 20 and 39 years. In our study in Austria, trends of decreasing incidence rates in all sex and age groups between 2013 and 2015 were observed, but the pattern ceased thereafter up to 2017. In women, incidence tended to increase in the last years except for the oldest subgroup.

An increased incidence of early onset type 2 diabetes was recently recognized as a growing global health problem in a systematic analysis of the Global Burden of Disease Study^13^. Moreover a greater disease burden in women aged < 30 years was evidenced. Another study^14^ showed high risk of early onset type 2 diabetes in women with all degrees of glucose intolerance during pregnancy which is the most frequent complication in pregnancy also in Austria^15^. Unfortunately we cannot report on possible trends of early onset type 2 diabetes or hyperglycemia during pregnancy in Austria because only patients older than 50 years were included in this analysis. Therefore, it can be assumed that the real number of incident diabetes cases are underestimated by our current analysis. Nevertheless type 2 diabetes is still rare in children or adolescents in Austria^1^, contrary to current trends of youth-onset type 2 diabetes in the USA, especially in ethnic minorities, or China^16^. Such trends are alarming as early onset diabetes was shown to be associated with higher risk of developing early complications of diabetes.

Note that in our analysis we would have observed increasing trends in incidence in the population older than 50 years, if we would have classified patients as incident instead of erratic upon treatment reinitiation, as we explored in an alternative model formulation we referred to as "NIDX model". Such measurements that do not adjust for these erratic patients would result in a slight but significant increase in incidence over time (max. 1.2%). Thus, this modeling analysis gives important new insights in diabetes care with potential impact on health policies. Comparing the erratic group with patients with diabetes on continuous antihyperglycemic treatment revealed that there was a predominance of females in the erratic group. Although we do not have an explanation for this finding it could be speculated that women stopped medication more often because they suffered more side effects of drug therapies or that they prefered to try lifestyle modification alone instead of additional drug therapies^15^. Furthermore, erratic patients appear healthier as they less often exhibit common risk factors like hypertension, dyslipidemia or obesity and diabetes-related important comorbidities like cardiovascular or renal diseases and cardiometabolic medication. Psychological diseases and depression were also more often diagnosed in the non-erratic group. However as comorbidities were derived from hospital stays during the observation period, an underestimation of all comorbidities can be supposed. This underestimation may particularly exist in the erratic group, possibly due to less health problems or less help seeking behavior or less accepting medical care. Although we cannot clarify this point the lower number of comorbidities may be ascribed to better health status of the erratic patients leading to lower drug adherence or clinical inertia. There could also be changes of the treatment plan with focus on lifestyle only, suspending medication. This would be plausible if glycemic control was improved because of weight reduction or stopping medication that counteracts insulin or diminishes insulin secretion (e.g. glucocorticoids, diuretics) or due to (temporary) resolution of a diabetogenic condition (e.g. severe illness, endocrine diseases). However, the rate of glucocorticoid prescription was even somewhat lower in the erratic group. Furthermore, it is well known that patients predisposed to diabetes commonly show deterioration of metabolism with increasing age and therefore could need antihyperglycemic medication again after some time. The fact that we observed higher mortality rates in the erratic group than in patients on regular medication is alarming and contradicts the hypothesis of better health status of this specific group. This observation is concerning and deserves further investigations.

There are several explanations for the increase in incidence rates from 2015 to 2017: next to the natural degree of fluctuations this observation could be ascribed to the fact that at higher age many patients with undiagnosed diabetes are detected in hospital because of diabetes-related complications like myocardial infarction, stroke or kidney disease. We know that e.g. one third of patients with myocardial infarction have hyperglycemia which was unknown before the event^17–19^.

Our study is subject to several limitations. We identified diabetes patients based on dispensals of antihyperglycemic medications and number of HbA1c measurements only. Information on their blood glucose or HbA1c levels were not available, neither was diagnoses information from extramural care or outpatient visits available in Austria. Prescriptions for medications below a cost of EUR 4,70 were also not contained in the data, which, however, should not effect the antihyperglycemics considered in this work. There was also no information on socio-economic indicators or other clinical parameters, such as body mass index, smoking status, quality of glycemic control, and lifestyle factors like nutrition and exercise. About 60,000 patients had to be removed due to missing information on antidiabetic treatments, which might also bias our results.

We used a dataset curated by Austrian health agencies ("LEICON”) that is also subject to several limiations due to its construction, see also^1,7,8^. Diabetes patients were identified through drug prescriptions or HbA1c measurements as there is no standardized ICD-10 coding in the Austrian outpatient sector, meaning it is not possible to directly distinguish type 2 diabetes from other types of diabetes. In particular, patients below an age of 50y that received insulin prescriptions (ATC codes starting with A10A) were excluded in the LEICON dataset due to the higher likelihood of type 1 diabetes in younger diabetes patients. We, therefore, restrict our analysis to patients aged > 50 y. A validation analysis found that the LEICON dataset identified type 2 diabetes patients with sensitivities and specificities above 80%^1,7,8^.

In conclusion, incidence of type 2 diabetes strongly depends on age, sex and place of residency. Correcting for erratic patients with diabetes, a slight decrease in total diabetes incidence and a concomitant increase in diabetes prevalence was observed, probably owing to an aging population. The large group of patients who fluctuate on and off of antihyperglycemic therapy and monitoring deserves further studies.

## Supplementary Information

Supplementary Information.

## Data Availability

Requests for accessing the data should be directed to Gottfried Endel under gottfried.endel@sozialversicherung.at. Custom code written for the analysis is available upon request to Peter Klimek, peter.klimek@meduniwien.ac.at.
