# Supplementary material for: Trends in diabetes incidence in Austria 2013–2017
Source: Sci Rep. 2023 May 29;13:8715. doi: 10.1038/s41598-023-35806-0 (PMC10227037; doi:10.1038/s41598-023-35806-0)
Supplement: Supplementary file 1 — Supplementary Information. [file 41598_2023_35806_MOESM1_ESM.pdf]

Trends in Diabetes Incidence in Austria 2013–2017

Michaela Kaleta, Michael Leutner, Stefan Thurner, Gottfried Endel, Noemi Kiss, Martin Robausch, Peter Klimek, Alexandra Kautzky-Willer

Supplementary Material

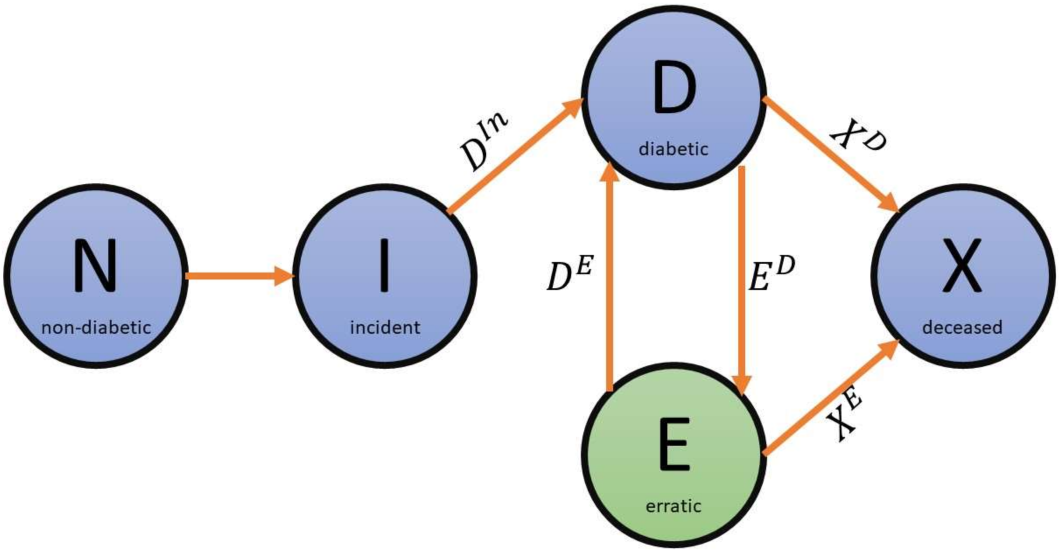

Figure S1. Overview of the NIDEX model to estimate diabetes incidence rates. Non-diabetes patients turn incident with a given rate and assume a diabetes state after receiving diabetes treatments. If these treatments are suspended for more than one year, they transition to the erratic state, from which they might return to the diabetes state if treatment is resumed. Patients die with different rates in the diabtes and erratic state.

Modelling incidence rates

In the NIDEX model, see Figure S1, the total number of diabetes patients with age (or age groups) a, sex g in year t, $D(a,t,g)$ were estimated, based on the number of non-deceased diabetes patients in the last year, $D(a-1,t-1,g)$, in addition to all inflows to and outflows from the diabetes state. Inflows are the number of incident patients from the non-diabetes state, $D^{In}(a,t,g)$, and the erratic state, $D^E(a,t,g)$. Outflows are given by the number of

excident patients, E^D, and the number of deceased patients with diabetes, $X^D(a-1,t-1,g)$. This gives the following stock-flow equation for the age, sex and time dependent number of patients with diabetes,

$$D(a,t,g) = D(a-1,t-1,g) + D^{In}(a,t,g) + D^E(a,t,g) - E^D(a,t,g) - X^D(a-1,t-1,g). \quad (1)$$

The number of erratic patients can be expressed in a similar stock-flow equation. Using the number of deceased erratic patients, $X^E(a-1,t-1,g)$, we have

$$E(a,t,g) = E(a-1,t-1,g) - D^E(a,t,g) + E^D(a,t,g) - X^E(a-1,t-1,g). \quad (2)$$

From this model, dynamical age and sex dependent incidence rates can be derived. Let $N(a,t,g)$ be the total population of age *a* and sex *g* in year *t*. The diabetes incidence rate $\alpha(a,t,g)$ is the fraction of incident patients out of the general population which is not in a diabetes or erratic state,

$$\alpha(a,t,g) = D^{In}(a,t,g)/[N(a-1,t-1,g) - D(a-1,t-1,g) - E(a-1,t-1,g)]. \quad (3)$$

Analogously, one could define an incidence rate $\alpha_E(a,t,g)$ from the erratic state, $\alpha_E(a,t,g) = D^E(a,t,g) \,/\, E(a-1,t-1,g)$, or an excidence rate $\beta(a,t,g)$ describing transitions from diabetes to erratic, $\beta(a,t,g) = E^D(a,t,g) \,/\, [D(a-1,t-1,g) - X^E(a-1,t-1,g)]$. We do not explicitly model mortality of the general non-diabetes population.

To obtain age and sex standardized incidence rates [1], age groups of ten years for each sex were considered. Thereby the regional diabetes populations were standardized to the general Austrian population. Directly standardized rates $\alpha_S(a,t,g)_B$ are used to correct for effects of age, sex and others and are estimated for each district B as $\alpha_S(a,t,g)_B = (\sum_{a,g} \frac{D^{In}(a,t,g)_B}{P(a,t,g)_B} * P(a,t,g))/(\sum_{a,g} P(a,t,g))$. The $\frac{D^{In}(a,t,g)_B}{P(a,t,g)_B}$ are the group specific rates of incident patients D^{In} in the district population $P(a,t,g)_B$ and $P(a,t,g)$ is the total population of age a, sex g in year t. We compare the study population in districts to the entire Austrian population as a standard population. Analogous standardisation is applied in the prevalence analysis by replacing the group specific incidence with prevalence.

Alternative model for incidence rates

The findings are compared with a dynamic model that does not contain an erratic patient state. In terms of the state dynamics shown in Figure S1, in this NIDX model the erratic state along with its in- and outflows were removed. Therefore, a flow $D^{-}(a,t,g)$ from $D(a,t,g)$ to the non-diabetes general population was introduced. We obtain a different incidence rate, $\delta(a,t,g)$, given by $\delta(a,t,g) = D^{In}(a,t,g)/(N(a-1,t-1,g) - D(a-1,t-1,g))$. There are two differences with respect to the incidence rate in Eq. 3. First, patients with diabetes might transition back to the non-diabetes state after discontinueing their treatment and thereby be counted as incident twice (the numerator $D^{In}(a,t,g)$ will in general be higher in the NIDX as in the NIDEX model). Second, the erratic patients of the NIDEX model are included in the general non-diabetes population of the NIDX model (larger denominator in the latter).

Analogous to the NIDEX model, the number of deceased patients with diabetes in the previous year is given by $X^D(a-1,t-1,g)$, the number of patients leaving the diabetes state is $E^D(a,t,g)$. The number of patients in the diabetes state in the new year $D(a,t,g)$ is then in turn dependent on previous year's D and X^D and the number of incident patients D^{In} and excident patients E^D in the present year and can be estimated as $D(a,t,g) = D(a-1,t-1,g) - X^D(a-1,t-1,g) + D^{In}(a,t,g) - E^D(a,t,g)$.

Baseline characteristics of patients with diabetes and erratic patients

	Diabetes cohort	Erratic cohort	p-value
N	477504	268680	<0.01
Age 2012 (mean +/- SD)	67±12	67±11	/
Sex (females,%)	230654 (48%)	140960 (52%)	<0.01
Major depressive disorder (F32-F33)	23060 (4.8%)	8405 (3.1%)	<0.01
Dementia (F03)	14252 (3.0%)	3581 (1.3%)	<0.01
Dementia in Alzheimer's disease (F00)	4499 (0.94%)	1279 (0.48%)	<0.01
Arterial hypertension (I10)	191659 (40%)	61811 (23%)	<0.01
Hyperlipidemia (E78.5)	37559 (7.9%)	10255 (3.8%)	<0.01
Ischemic heart diseases (I20–I25)	87199 (18%)	25408 (9.5%)	<0.01
Myocardial infarction (I21.09+I21.3+I25.2)	6908 (1.4%)	1604 (0.60%)	<0.01
Stroke (I63, I64)	21197 (4.4%)	5134 (1.9%)	<0.01
Heart failure (I50)	43954 (9.2%)	10157 (3.8%)	<0.01
pAVK (I73.8+I73.9)	25061 (5.2%)	5206 (1.9%)	<0.01
Acute kidney failure and chronic kidney disease (N17-N19)	63205 (13%)	15184 (5.7%)	<0.01
Fatty liver (K76.0)	17407 (3.6%)	4537 (1.7%)	<0.01
Fibrosis and cirrhosis of liver (K74)	4990 (1.0%)	1135 (0.42%)	<0.01
Nicotine dependency (F17)	14632 (3.1%)	4051 (1.5%)	<0.01
Alcohol abuse (F10.1)	1959 (0.41%)	659 (0.25%)	<0.01
Polycystic Ovary Syndrome (E28.2)	13 (0.0027%)	2 (0.00074%)	0.12
Dependence on renal dialysis (Z99.2)	616 (0.13%)	137 (0.051%)	<0.01
Heart transplant status (Z94.1)	155 (0.032%)	37 (0.014%)	<0.01
Kidney transplant status (Z94.0)	716 (0.15%)	207 (0.077%)	<0.01
Liver transplant status (Z94.4)	258 (0.054%)	65 (0.024%)	<0.01
St.p. surgery (Z98)	5568 (1.2%)	1847 (0.69%)	<0.01
Eating disorders (F50)	255 (0.053%)	65 (0.024%)	<0.01

Osteoporosis (M80–M82)	17960 (3.8%)	7386 (2.7%)	<0.01
Diseases of arteries (I70–I79)	47779 (10%)	11601 (4.3%)	<0.01
Overweight and obesity (E66)	44667 (9.4%)	12322 (4.6%)	<0.01
COPD (J44)	32633 (6.8%)	10050 (3.7%)	<0.01
Arthritis (M06)	2831 (0.59%)	1368 (0.51%)	<0.01
Insulin (A10A)	113917 (24%)	11713 (4.4%)	<0.01
Oral antihyperglycemics (A10B)	361385 (76%)	91822 (34%)	<0.01
Biguanides (A10BA)	262090 (55%)	69187 (26%)	<0.01
Sulfonylureas (A10BB)	156017 (33%)	23759 (8.8%)	<0.01
Combinations of oral blood glucose lowering drugs (A10BD)	137295 (29%)	14884 (5.5%)	<0.01
Alpha glucosidase inhibitors (A10BF)	5854 (1.2%)	917 (0.34%)	<0.01
Thiazolidinediones (A10BG)	27567 (5.8%)	2342 (0.87%)	<0.01
DPP4-inhibitors (A10BH)	88274 (18%)	10424 (3.9%)	<0.01
GLP-1 analogues (A10BJ)	6499 (1.4%)	234 (0.087%)	<0.01
SGLT2-inhibitors (A10BK)	32769 (6.9%)	2401 (0.89%)	<0.01
Other blood glucose lowering drugs, excluding insulins (A10BX)	34861 (7.3%)	3470 (1.3%)	<0.01
Fibrates (C10AB)	21833 (4.6%)	8333 (3.1%)	<0.01
Lipid modifying agents (C10A)	303076 (63%)	135389 (50%)	<0.01
Statins (C10AA)	294930 (62%)	130712 (49%)	<0.01
ARB (C09CA)	99083 (21%)	46432 (17%)	<0.01
Beta blocking agents (C07)	227415 (48%)	100328 (37%)	<0.01
Aspirin (B01AC06)	122665 (26%)	36045 (13%)	<0.01
Proton pump inhibitors (A02BC)	294780 (62%)	145752 (54%)	<0.01
Aldosterone antagonists (C03DA)	51943 (11%)	17427 (6.5%)	<0.01
Clopidogrel (B01AC04)	58185 (12%)	20223 (7.5%)	<0.01
Ticagrelor (B01AC24)	5973 (1.3%)	1931 (0.72%)	<0.01
Prasugrel (B01AC22)	3422 (0.72%)	1300 (0.48%)	<0.01
Antidepressants (N06A)	162611 (34%)	76848 (29%)	<0.01
Glucocorticoids (H02AB09)	659 (0.14%)	327 (0.12%)	0.068

Corticosteroids (H02)	123686 (26%)	54384(20%)	<0.01

Table S1. Group-based comparison between the diabetes cohort and the erratic cohort. Case numbers were counted between 2012 and 2017.

Pearson's correlation

Pearson's correlation tests were used after standardizing for age and sex to determine correlation between incidence rates and prevalence and ultimately to compare modelling results. Each model is represented by a vector containing standardised incidence rates/prevalence for each district in Austria as described by the $\alpha_S(a,t,g)_B$. The correlation can be tested for each year, Table S1 shows the correlation coefficients of 2016 according to results shown in the regional variation (see Figure S4).

	δ	α	prevalence
δ	1.000	0.983	0.874
α	0.983	1.000	0.830
prevalence	0.874	0.830	1.000

Table S2. Pearson's correlation coefficients for standardised results of 2016 to compare the NIDEX model (α) to the NIDX model (δ) and the general prevalence.

References

[1] Schoenbach, V. J. & Rosamond, W. D. *Understanding the fundamentals of epidemiology: an evolving text*. http://www.epidemiolog.net/evolving/ (2000).
